# Implications of the Nucleocapsid and the Microenvironment in Retroviral Reverse Transcription

**DOI:** 10.3390/v2040939

**Published:** 2010-04-02

**Authors:** Marylène Mougel, Andrea Cimarelli, Jean-Luc Darlix

**Affiliations:** 1 CPBS, UMR5236 CNRS, UMI, 4 bd Henri IV, 34965 Montpellier, France; E-Mail: mmougel@univ-montp1.fr; 2 LaboRetro Unité de Virologie humaine INSERM #758, IFR128, ENS Lyon, 46 Allée d’Italie, 69364 Lyon, France; E-Mail: acimarel@ens-lyon.fr

**Keywords:** reverse transcriptase, nucleocapsid protein, genomic RNA, viral DNA, strand transfers, SEVI

## Abstract

This mini-review summarizes the process of reverse-transcription, an obligatory step in retrovirus replication during which the retroviral RNA/DNA-dependent DNA polymerase (RT) copies the single-stranded genomic RNA to generate the double-stranded viral DNA while degrading the genomic RNA via its associated RNase H activity. The hybridization of complementary viral sequences by the nucleocapsid protein (NC) receives a special focus, since it acts to chaperone the strand transfers obligatory for synthesis of the complete viral DNA and flanking long terminal repeats (LTR). Since the physiological microenvironment can impact on reverse-transcription, this mini-review also focuses on factors present in the intra-cellular or extra-cellular milieu that can drastically influence both the timing and the activity of reverse-transcription and hence virus infectivity.

## 1. Retroviruses as mobile elements in nature

A unique feature of Retroviruses concerns their replication mechanism in host cells and organisms, a reverse transcription-integration process whereby the single-stranded viral RNA is copied by the viral DNA polymerase to generate the proviral DNA, which is integrated into the host cell DNA [1–9]. A genetic insertion such as this can impact on the life of the host and in some instances that of its descendants. In that respect, Retroviruses appear to be unique since no other infectious agent of higher eukaryotes is capable of integrating its genes into the host genome, of acquiring cellular genes into its own genome or has played such seminal roles in modern biology, biotechnology and in gene therapy [10–13].

**Figure 1.**
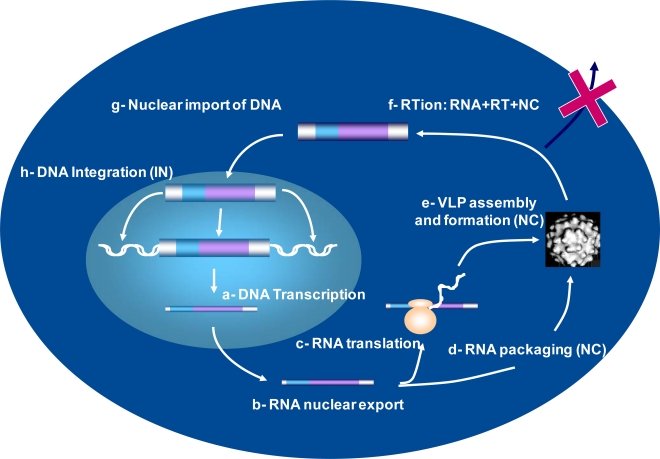
**Schematic representation of the replication of a simple retrotransposon. a.** The genomic RNA is a unique RNA synthesized by transcription of the integrated retrotransposon DNA. **b.-c.** The RNA copy is exported from the nucleus and translated by the cellular translation machinery – ribosomes are illustrated here - to produce the Gag and GagPol like polyprotein precursors. **d.-e.** During formation of a ribonucleoparticle (RNP) called VLP (virus-like particle or VLP) the Gag and GagPol precursors undergo maturation by a Pol-encoded protease. At the same time the RNA copy of the retrotransposon, together with the replication primer tRNA are incorporated into the VLP. Note that the VLP’s remain in the cytoplasm and are not exported (cross) contrary to replication-competent retroviruses. **f.** Reverse transcription of the RNA copy is carried out by the RT and is chaperoned by NC-like proteins in the VLP nucleoprotein structure to generate a new copy of retrotransposon DNA. **g.-h.** The new DNA copy is imported into the nucleus and integrated into the host cell genome by the Pol-encoded integrase to complete the copy-and-paste process.

Among Retroelements, the LTR-containing retrotransposons such as yeast retrotransposons (Ty) and human endogenous retroviruses (HERV) resemble simple retroviruses such as the gammaretrovirus murine leukemia virus (MuLV). In fact, these mobile elements encode the virion structural Gag proteins and Pol enzymes and contain non-coding regulatory sequences essential for genome replication, integration and expression such as the LTR (long terminal repeats). The endogenous retrotransposons that are abundant genetic elements in the host genetic make-up are probably playing key roles in genome reshuffling and variability. Thus, the replication of mobile retrotransposons by a transcription/reverse-transcription/integration process, also called ‘copy-and-paste’, is thought to have fueled the evolution of eukaryotic genomes from yeast to human (Figure 1 and legend) (reviewed in [14–17]).

Retroviral particles or virions have a globular structure with a mean diameter of 100 nanometers. The virion outer envelope is of cellular origin and contains the viral glycoproteins called surface (SU) and transmembrane (TM), in the form of trimeric ensembles for HIV-1. The shape and oligomeric organization of the structural proteins of the inner core seem to be characteristics for each genera of the *Retroviridae* family. The virion genomic RNA is 6,000 to 12,000 nucleotides in length with a positive polarity, and resides within the core as a 60S RNA complex, where two full length viral RNA molecules interact with each other and are coated by several hundred nucleocapsid protein molecules (about 1500 molecules in the case of HIV-1 and MuLV [18–21]).

Furthermore, it is important to briefly point out that actively replicating retroviral populations can have a complex composition, containing notably defective retroviruses which replicate only with the help of a replication competent retrovirus, called helper [4]. In fact, defective retroviruses commonly found in retroviral populations, need part or all of the functions of a fully competent retrovirus to replicate and disseminate in cells and organisms. Canonical defective retroviruses include highly oncogenic DLV’s (defective leukemia viruses) such as the Harvey and Kirsten MSV, which carries the v.ras oncogene flanked by retrotransposon VL30 sequences. The Moloney murine leukemia virus (MoMuLV) provides the viral helper functions in *trans* within the same cell to ensure co-replication of these DLV’s (see [22] for review).

## 2. Reverse transcription of the genomic RNA

### Forewords

The reverse transcription reaction, whereby the positive strand genomic RNA serves as the template for the synthesis of a double-stranded DNA flanked by long terminal repeats (LTR), occurs during the early phase of virus replication, soon after virus infection of a target cell. The process of viral DNA synthesis by RT initially takes place in the virion core after its entry into the cytoplasm. The virion core is thought to undergo structural changes to become the reverse transcription complex (RTC; review of [20,21,23–25]).

We will begin by providing a brief overview of viral DNA synthesis, from initiation to completion, with an emphasis on the interplay between RT, the viral nucleic acids and the nucleocapsid protein, the latter of which is an essential viral cofactor for viral nucleic acids and the RT enzyme. We will continue by reviewing factors that are believed to have an impact on reverse transcription, its fidelity as well as the potential variability of virus progeny. Finally, we will discuss recent findings on when, where and how reverse transcription takes place.

#### a- Simplified scheme of the viral RNA template

**Figure 2.**
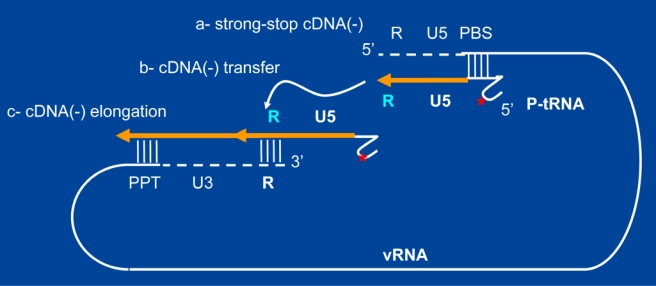
***In vitro* model systems to study retroviral reverse transcription.** Flexible *in vitro* model systems have been set up to study in detail the process of retrovirus reverse transcription [21,23–25,30]. Such models include (i) *in vitro* generated RNA (vRNA) representing the 5′ and 3′ UTR domains containing the *cis*-acting elements essential for cDNA synthesis, namely the PBS, the binding site for the replication primer tRNA, the untranslated 5′ and 3′ regions (U5 and U3), the repeats (R in blue) and the polypurine tract (PPT); (ii) Replication primer tRNA of natural origin (P-tRNA) or generated by *in vitro* transcription, or a synthetic oligonucleotide complementary to the PBS; (iii) the RT enzyme (not shown); (iv) NC protein (not shown); (v) if required, the IN enzyme, VIF, VPR and cellular factors such as SEVI [31]. *In vitro* models such as this have rendered possible a detailed investigation of the essential steps of reverse transcription, following tRNA annealing to the PBS by NC:
a- initiation of ss-cDNA synthesis (see large orange arrow);b- the first strand transfer which corresponds to an annealing reaction chaperoned by NC and requiring the R sequences (white arrow) [31–32];c- minus-strand cDNA elongation (double orange arrow);d- initiation of plus-strand DNA synthesis and transfer (not shown here for the sake of clarity; see also Figure 3);e- the fidelity of the strand transfer and of cDNA synthesis by RT and the influence of RT mutations;f- the role of the RT-associated RNase H activity on the strand transfer;g- the role of NC on DNA strand transfer and the fidelity of reverse transcription via its interaction with RT and the vRNA;h- the influence of vRNA mutations, incubation conditions (ions, temperature, nucleotides *etc.*) and viral and cellular factors such as VIF, SEVI.

**Figure 3.**
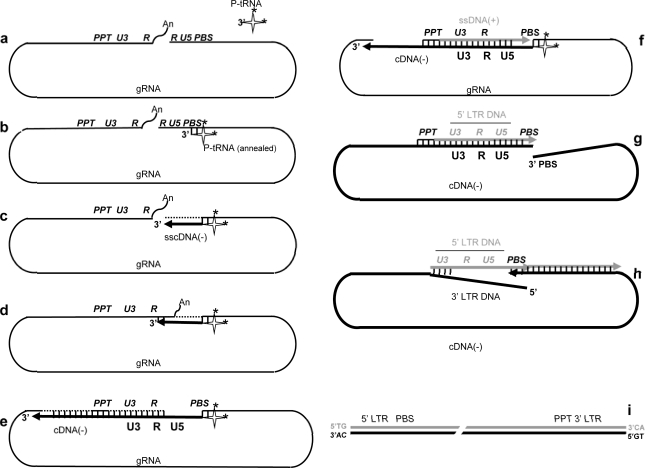
**Illustration of the reverse transcription process.** The individual steps are as follows. **a.-b.** annealing of the replication primer tRNA by NC. Stars correspond to modified nucleotides in the primer tRNA, notably m6A at position 58 important for the fidelity of the plus-strand DNA transfer and in the anti-codon loop recognized by RT. **c.** Initiation of cDNA synthesis by RT by extension of the –CCA 3′ terminal nucleotides. **d.** SscDNA(−) transfer to the RNA 3′ R sequences by NC. **e.** minus -trand DNA transfer by RT. **f.** Initiation of plus-strand DNA by extension of the PPT RNA by RT. **g.-h.** Plus-strand DNA transfer at the level of the PBS sequences by NC and elongation of viral DNA strands by RT that includes ds DNA unwinding to complete LTR DNA synthesis. **i.** The linear ds DNA is shown here with the LTR’s and the terminal TG/CA nucleotides.

The 5′ and 3′ untranslated regions (UTR) of the full-length viral RNA are highlighted in Figure 3 since they contain signals essential for reverse transcription from initiation to completion. The UTRs are made up of functional units referred to as R, U5 and PBS (5′ UTR), and PPT, U3, R and An (3′ UTR). Abbreviations stand for the Repeats (R), the untranslated 5′ and 3′ sequences (U5 and U3), the tRNA primer binding site (PBS), the polypurine tract (PPT) and the 3′ polyA tail (wavy line illustrating the 3′ polyA tail) [26]. The cellular primer tRNALys,3 is represented by a cloverleaf-like molecule where modified bases (such as m6A at position 58 (see below)) are highlighted by black stars.. Although the genomic RNA is dimeric in a condensed 60S form within virions, only a single retroviral RNA molecule (gRNA) is shown here as a pseudo-circle, where the 5′ and 3′ ends are in close proximity. Note that the viral DNA polymerase (RT) and the NC protein molecules, which coat the genomic RNA, cannot be represented in this highly schematic flow diagram. Our understanding of reverse transcription has largely benefited from *in vitro* model systems to study the major steps of viral DNA synthesis (Figure 2).

#### b- Primer tRNA annealing

In most retroviruses the PBS is 18 nt in length and annealing of replication primer tRNA, - tRNALys,3 for Lentiviruses - via its complementary 3′ 18 nt is promoted by means of the RNA annealing activity of NC proteins. This RNA annealing activity is exhibited by both the polyprotein Gag precursor and the mature NC protein (reviewed in ([27]) and results in the formation of a perfect 18 nt long double-stranded RNA between the viral PBS and primer tRNA (Figure 3a, b). Further nucleotide interactions have been reported to take place between tRNALys,3 and motifs flanking the PBS in HIV-1, notably the A rich polyA stem-loop [25–27].

#### c- Initiation of viral DNA synthesis

RT recognizes the terminal 3′ OH of the annealed tRNA to initiate reverse transcription and synthesis of the minus-strand strong-stop cDNA (sscDNA) by copying the genomic RNA (thick black line) (Figure 3c). Other factors have been found to contribute to RT recruitment onto the viral initiation site such as tRNA nucleotide modifications at the level of the anti-codon loop and NC protein (reviewed in 21). At the end of sscDNA synthesis, the 5′ gRNA sequences exist in a DNA:RNA hybrid and undergo degradation by the RT-associated RNaseH activity [28] (reviewed in [29]) resulting in the formation of a single-stranded DNA covalently linked to the primer tRNA. The RNase H activity appears to be enhanced by NC protein in the course of and following vDNA synthesis, probably by promotion of the release of small RNA degradation fragments generated by RNase H [29–30].

#### d- Transfer of sscDNA

The first DNA strand transfer corresponds to a hybridization reaction between the ss-cDNA 3′ R(-) and gRNA 3′ R(+) sequences, which is required to resume reverse transcription of the gRNA and to synthesize the 3′ LTR. This hybridization reaction between the R(-) and R(+) sequences is directed by NC protein [30,32] (reviewed in [20–21]) According to *in vitro* analysis on HIV-1, it appears that both the TAR upper loop and the R ends are important for this annealing reaction [33] (Figure 3d).

#### e-f- Minus strand cDNA synthesis

The minus-strand sscDNA is extended by RT by copying the gRNA up to the 3′ end of the PBS while at the same time the genomic RNA template is being degraded by the RT-RNase H activity (Figure 3e). Interactions between the RT enzyme and NC protein molecules augment the processivity of the reaction *in vitro* as well as its fidelity by providing a degree of excision-repair activity to RT in HIV-1 [20,34] and MuLV (Darlix *et al.*, unpublished data).

#### g- Initiation of plus-strand DNA synthesis

The plus-strand primer or polypurine tract (PPT) directs initiation of plus-strand DNA synthesis as a result of a sequential process: (i) minus-strand DNA synthesis over the PPT, (ii) RT-RNase H cleavage at the PPT 3′ end, which allows initiation (iii) of plus-strand DNA synthesis from the nascent RNA primer (Figure 3f) with the help of NC protein [35,36].

Later in the course of reverse transcription the PPT RNA is removed by the RT-RNase H activity. RT synthesizes plus-strand DNA (gray line) [37–38] by extension of the genomic PPT RNA and continues up to the methylated A residue at position 58 of primer tRNALys,3 [38]. This results in the formation of a double-stranded DNA encompassing the U3, R and U5 sequences, corresponding to the full-length 3′ LTR and in the release of the remaining tRNALys,3 sequences by the RT RNaseH activity, freeing the 3′ end of the newly made plus- and minus-strand DNA.

#### h- Plus-strand DNA transfer

This step corresponds to a hybridization reaction between the minus- and plus-strand viral DNA at the level of the PBS sequences (see ref. [39] for detail), in a reaction directed by NC protein *in vitro* (Figure 3g, h)(reviewed in [20–21]). Subsequently the two viral DNA strands are extended by RT, the plus-strand by copying the newly made minus-strand viral DNA, on the one hand, and the final extension of the minus-strand viral DNA by copying the plus-strand DNA, which requires DNA strand displacement, ultimately leading to 5′ LTR formation, on the other hand.

#### i-j- The proviral DNA

A double-stranded linear DNA with the two flanking LTR’s is the final product of the reverse transcription process and its maintenance, notably of the inverted repeats (ir) that are required for integration by the viral integrase enzyme (IN), is ensured by NC protein and IN molecules (Figure 3i) [40–41] (reviewed in [20]). This viral DNA, also called proviral DNA, is actively imported into the nucleus within a preintegration complex (PIC) and is subsequently integrated into the host cell genome by IN in the form of a tetramer with the help of the cellular cofactor LEDGF [8,42–43]. Recently developed anti-HIV-1 drugs were found to efficiently inhibit the integration reaction *in vitro* and *in vivo* [44].

## 3. Reverse transcription of subgenomic and cellular RNAs

The full length viral RNA, in addition to being the retroviral genome, plays a key role early in retrovirus assembly since it represents a scaffolding platform onto which Gag polyprotein molecules readily assemble to ultimately form the inner core structure [45–47]. In addition to the dimeric RNA genome, which is the reverse transcription template, the mature virion core structure contains subgenomic and a subset of cellular RNAs that can be reverse transcribed.

### 

#### a. Virion incorporation of subgenomic viral RNAs

Significant amounts of subgenomic viral RNAs are packaged into virions alongside the full length viral RNA [49–51]. Under conditions where the full length viral RNA has had the Psi packaging signal removed, only minute amounts of genomic RNA are incorporated into virions, while the incorporation of subgenomic RNA is favored (for review : [52]) [49,53–55]. The spliced viral RNAs are believed to be dimeric within the virion core since they are able to undergo dimerization *in vitro*, a prerequisite for RNA packaging [51,56–57]. All spliced HIV-1 RNA species have been found equally packaged in virions, regardless of their nuclear export pathway [49]. While genomic RNA packaging is mediated through Gag or NC interaction, involving the characteristic zinc finger domains, the spliced HIV RNAs are dependent upon the SP1 region of Gag for their packaging [54]. However, all HIV-1 RNA species share common functional *cis*-acting packaging signals, notably those in the 5′ UTR, since both the genomic RNA and the spliced viral RNA species are packaged in a competitive manner [49]. The subgenomic RNAs contain all the *cis*-acting signals recognized by RT, and reverse transcription of notably the fully spliced viral RNAs takes place in virions and in newly infected cells as efficiently as that of the unspliced viral RNAs [50]. In the case of gammaretroviruses, MuLV provides the unique example of a natural heterodimer. Despite the presence of spliced and genomic RNA within virions, genomic dimers are the predominant form observed undergoing reverse transcription [51,58].

#### b. Incorporation of cellular RNAs into virions

In the course of virion morphogenesis, abundant cellular RNAs are incorporated into viral particles, which, in the case of wild-type retroviral particles, reside in the core together with the genomic RNA. Interestingly, incorporation of cellular RNAs into viral particles is greatly enhanced when the genomic RNA lacks the Psi packaging signal. Under these conditions, cellular RNAs can constitute more than 50% of the total RNA mass per particle [46,49]. These cellular RNAs might participate in recombination-transduction reactions during reverse transcription, for example defective leukemia viruses have been generated through this process [59–64].

Figures vary for the extent of cellular RNA incorporation into virions since RNA can be selectively excluded or enriched for in retroviral particles. Among abundant species, there are many small Pol-III-generated RNAs such as tRNA, 5S rRNA, U6 snRNA, mY RNAs, 7SL RNA and 7SL RNA-derived SINE RNAs (B1 RNA or Alu RNA) [49,64–68]. For unknown reasons, the U6 snRNA is enriched in RSV, MuLV or HIV-1 particles, while U1 and U2 snRNAs are found only in trace amounts.

The 7SL RNA exemplifies specific host RNA encapsidation since it is enriched at a level similar to that of the genomic RNA in MuLV and HIV-1 virions [65–66]. In addition, the 7SL RNA can be reverse transcribed in virions and in newly infected cells [67].

Similarly, mY RNAs are among the most highly represented non-coding RNAs specifically packaged in MuLV. Interestingly, mY RNAs are recruited even in Ro60 knockout cells, where mY RNA is degraded in the cytoplasm while there is a residual pool in the nucleus [68–69]. These observations favor the notion that mY RNAs are recruited in the nucleus of infected cells, at an early stage of MuLV morphogenesis. Except for the replication primer tRNAs that are packaged through interaction with viral RT and possibly NC [70–72], the packaging determinants of host cell RNAs are poorly defined. Recent studies have indicated that selective packaging of host RNAs such as U6 snRNA or 7SL RNA, is controlled through independent mechanisms that differ from those of viral RNAs [49,64,66]. A possible mechanism through their heterodimerization with the viral genomic RNA is unlikely [51]. Packaging of 7SL RNA has been studied the widest, but its NC-dependence, and its possible influence on APOBEC3G packaging are questioned by others [73].

Taken together, the mechanism by which non-genomic, viral and cellular RNAs are packaged into virions is as yet poorly understood, or controversial, with the exception of the primer tRNA. The roles of subgenomic RNA and some cellular RNAs in reverse transcription and in the biology of the virus remain a matter of speculation, with the notable exception of the replication primer, and of 7SL RNA, thought to recruit the APOBEC 3G restriction factor [70,74].

### 4. Viral DNA Synthesis during retrovirus assembly

The overall process of Retrovirus assembly is considered to take place at the plasma membrane of infected cells where Gag and Gag-Pol molecules assemble via major interactions between two platforms, the N-terminal myristate and basic residues of the Gag-Matrix with plasma membrane phospholipids, and the zinc finger and the flanking basic residues of the Gag-NC with the genomic RNA (reviewed in [75–79]). Once completed, immature retroviral particles bud from the plasma membrane. Next, virion Gag and Pol molecules are processed by the viral protease (PR) during which condensation of the inner core occurs and virions gain infectivity [80–81]. However, retroviral assembly can also take place within infected cells, notably at the level of intracellular membranes such as endosomes and multivesicular bodies [82–85]. Under these circumstances the viral protease can readily remain active during assembly, directing the processing of Gag and Gag-Pol polyprotein precursors. Indeed, a large body of evidence obtained by western immunobloting demonstrates the presence of mature matrix, capsid and nucleocapsid proteins in cytoplasmic extracts of infected cells. Furthermore, the newly made virions contain a single copy of the full length viral RNA in a dimeric form along with minor quantities of spliced RNAs (see above) [49].

However, it has long been shown that small amounts of minus-strand viral DNA are present in viral particles of Rous sarcoma virus (RSV), Moloney murine leukemia virus (MoMuLV), and HIV-1 generated in cell cultures. This indicates that reverse transcription can already be ongoing during virus assembly or in the released particles, in a process which has been called natural endogenous reverse transcription (NERT) [86–89]. This early or premature reverse transcription has been extensively studied by the groups of Pomerantz and Mougel [50,90], revealing that AZT treatment of HIV-1 infected T cells causes a 10–100 fold reduction of intravirion viral DNA levels. This supports the notion that reverse transcription can take place, at least in part during the course of virus assembly as suggested some time ago in studies demonstrating that the synthesis of a complete infectious viral DNA can take place within virions of MoMuLV and equine infectious anemia virus (EIAV) under *in vitro* conditions using mild detergents that preserve the core integrity [92–93].More importantly, the physiological microenvironment, notably components of seminal fluid such as polyamines, dNTP’s and fragments of the abundant semen marker prostatic acidic phosphatase (PAP)s, can drastically enhance NERT and the accumulation of intravirion viral DNA [31–90]. HIV-1 virions containing high levels of viral DNA can readily infect non-dividing target cells. In addition, the PAP-derived peptide, termed Semen-derived Enhancer of Virus Infection (SEVI) that is also abundant in seminal fluid can efficiently promote attachment of HIV-1 particles to target cells. This in turn enhances viral infection and most probably facilitates the very early events of HIV-1 infection during sexual intercourse [31,90,91].

Furthermore, SEVI seems to display a potent nucleic acid chaperoning activity and to greatly enhance the reverse transcription process *in vitro* as well as in HIV-1 virions (Darlix JL *et al.*, unpublished data), which suggests that SEVI might act as a multifunctional cofactor enhancing HIV-1 infection through numerous modes.

Taken together these findings show that viral DNA synthesis can indeed start during virus formation and that the so-called NERT process is largely influenced by components of the physiological microenvironment such as polyamines, dNTP’s and SEVI. This in turn can have a significant impact on HIV-1 infectivity of primary target cells [89–91].

Arguably, Retroviruses such as HIV should be viewed as RNA/DNA viruses rather than being strictly categorized as RNA viruses. Furthermore, the evidence highlights the need to identify microbicide compounds aimed at inhibiting components of the seminal fluid which facilitate HIV-1 infection [94].

### 5. Retrovirus assembly and the control of reverse transcription

In the course of assembly, Gag structural precursor molecules are targeted to the plasma membrane by the N-myristate and basic residues of the Gag matrix (MA) on the one hand, and the genomic RNA, notably the Psi Packaging signal (its stem-loops specifically bound by the C-terminal Gag nucleocapsid (NC)), on the other. In fact, NC – consisting of either one or two highly conserved zinc fingers flanked by basic residues – directs the selection of the genomic RNA through multiple interactions between the RNA Psi sequences and both its zinc finger(s) and its basic residues [20,21,75,95].

In the case of HIV-1 NC, the central globular domain corresponding to the two zinc fingers and the basic linker forms a hydrophobic platform which specifically binds the Psi stem-loops of the genomic RNA (reviewed in [21,75]). Mutating the highly conserved CCHC residues of the zinc fingers causes an impairment of genomic RNA packaging in newly formed virions. Interestingly, these CCHC mutations result in profound modifications of Gag trafficking in cells and in the production of viral particles that are completely replication defective (reviewed in [20,95]).

However reverse transcription does take place in CCHC mutant virions as evidenced by the accumulation of newly made viral DNA ([96–97], and ref. herein), which does not, however undergo integration. The observed reverse transcription occurs during assembly and prior to virion budding because addition of the nucleoside RT inhibitor (NRTi) AZT to the producer cells blocks accumulation of the viral DNA. This newly synthesized viral DNA, or at least part of it, was found to be functional since it was capable of promoting synthesis of the viral Tat transactivator and of LTR activation *ex vivo* [96].

The above data highlight the fundamental role of NC in virus assembly and reverse transcription, and possibly in the maintenance of the newly made viral DNA [40] (reviewed in [20,95]). In addition, there seems to be a tight connection between virus assembly and the start of reverse transcription where slowing down or modifying the assembly process would be expected to modify the timing of viral DNA synthesis. The data also favor the notion that minor structural modifications of the inner virion core would result in the intracellular instability of newly made viral DNA, notably at the 5′ and 3′ ends. Indeed, mutating the NC zinc fingers results in the formation of HIV-1 particles with a general globular structure and not a condensed cone-shaped structure as typified by the wild type virus [99], which result in degradation of the incoming virion core by cellular factors (see § on the synthesis of functional viral particles in primary cells, below).

In support of such a dynamic connection between virus assembly and budding, and viral DNA synthesis, Thomas *et al.* [98] reported that mutating the p6 PTAP motif within the Gag C-terminal domain slowed virion budding, an effect probably due to weakening of the interaction between Gag and the cellular transporter protein TSG101. At the same time, this causes extensive premature reverse transcription and the accumulation of newly made viral DNA in p6 mutant virions.

Thus, the general view that emerges suggests that the dynamics of the assembly-budding process exert tight control on the timing of viral DNA synthesis by RT, chaperoned by NC in the virion core. Furthermore, the global structure of the core, central to which the multiple interactions between NC molecules and the genomic RNA, would transiently function as a shield against cellular factors capable of degrading components of the viral core, which would otherwise prevent synthesis of a functional viral DNA. For highly replicating viruses such as HIV-1, the nature of the molecular interactions between the genomic RNA, tRNA, RT and NC, and between the viral DNA, NC and IN largely out competes the influence of host restriction factors such as APOBEC and TRIM proteins. In addition, these tight molecular interactions might well be indispensable for the synthesis of complete viral DNA containing mutations and reassortment of large regions by means of recombination reactions. This should confer, at least in part, resistance to immune responses and anti-retroviral therapies targeting the RT, PR and IN enzymes (HAART).

## 6. Reverse transcription in primary cells

In cells undergoing HIV-1 infection, the process of reverse transcription occurs in a densely populated environment, the cytoplasm. There, it is intimately linked to at least two other steps, namely uncoating, the process through which the viral core is reorganized, and trafficking towards the nucleus [100]. Given that reverse transcription occurs in this viral shell that serves several functions, it is not surprising that this process is intimately linked to the fate of viral cores. Indeed, mutations that affect the core major structural component, (*i.e.* the Capsid protein (CA)), almost invariably display a defect in viral DNA accumulation [100–103].

However, the relationship between HIV-1 reverse transcription and viral core reorganization is poorly understood. Specifically, it is not known whether uncoating occurs subsequent to the completion of viral DNA or if a reciprocal co-stimulation is established between the two processes. The former hypothesis was raised following evidence indicating that viral cores gained stability in the absence of the central polypurine tract -central termination sequence (cPPT-CTS) *cis*-element [104]. In this debated model, the three-stranded DNA flap formed in the neo-synthesized viral DNA, conveys the signal of end of reverse transcription which starts viral core restructuration and prepares it for the next step of the viral life cycle, *i.e*. nuclear import. This model is seducing as it finds parallels in other viruses whose capsids undergo profound changes in the proximity of nuclear pores that are required for the nuclear import of their genome [104–106]. Whether this is true for HIV-1 remains a matter of debate as biochemical evidence suggests that extensive viral core reorganization occurs either prior to or during reverse transcription [107–110] and cPPT-CTS mutants display rather moderate infectivity defects [111–113].

Why is the relationship between reverse transcription and uncoating important? We propose it is so to counteract in a timely fashion possible antiviral defenses that target viral cores. Viral cores are characteristic molecular signatures of Retroviruses and they are known to be targeted shortly after cell entry by antiviral factors. For example, the Tripartite motif protein 5 alpha (TRIM 5α) recognizes viral cores and destructures them, impacting on reverse transcription (108). If uncoating does not occur until the completion of viral DNA synthesis, then a structure identical to the one entering the cell will be present throughout the journey of the viral core across cytoplasm. If, instead, uncoating occurs progressively together with reverse transcription this characteristic complex will undergo change, as a consequence of which the viral structures sensed by cellular antiviral defenses will be markedly distinct.

It remains to be seen if TRIM5α or the Friend susceptibility virus gene 1 (Fv1) are unique factors or if other proteins exert similar functions, but it would not be surprising to find these characteristic retroviral structures to be the target of multiple factors. In this respect, by providing a signal for the ordered reorganization of viral cores, reverse transcription may protect the virus from attack within the cytoplasm environment simply by promoting shedding of the components through which viral cores are recognized.

The major difference between the two models outlined above on the relationship between core reorganization and reverse transcription is the length of time during which the initial viral core structure persists as such in the cytoplasm of infected cells. It is well appreciated that viral DNA completion varies greatly among target cells. For example, while the reverse transcription process takes only 4 to 8 hours in transformed cell lines such as HeLa cells, widely used to study viral infection, it can take up to 20–30 hrs in primary lymphocytes and macrophages or days in non-stimulated monocytes [114–116]. Thus, the extent of reverse transcription in different target cells is extremely variable and dependent on the activation status of target cells, the dNTP pool and on the plethora of cellular factors that are present in these cells at the moment of infection. In cells in which reverse transcription occurs slowly, antiviral defenses are more likely to recognize and counteract incoming virion cores.

Indeed a correlation between the extent of reverse transcription and the permissivity of primary cells to infection is widely reported. In the past, this has been hypothesized to be a consequence of poor availability of dNTPs in cells resistant to infection. Yet, more recent data indicates that RT functions correctly even at low dNTP concentrations, such as those normally found in restrictive cells including macrophages or monocytes [116,117]. It is thus conceivable that the correlation between the rate of reverse transcription and a cell’s susceptibility to infection is due to a protective effect of the viral core permitting more efficient reverse transcription and thus infection.

One of the major problems in studying the early phases of infection is the long appreciated observation that the vast majority of viral particles entering cells are non-infectious, as they do not result in the establishment of an integrated proviral DNA and are progressively lost over time. Only 1 in 8 viruses is thought to be infectious in established cell lines where infection is efficient [118] but may well be at least 1 in 100 particles in primary cell types such as macrophages and dendritic cells. The reasons for this are unclear at present and defects at the level of viral assembly cannot be excluded. Yet, an interesting hypothesis is that intracellular defenses are largely responsible for the generation of non-infectious viruses, in which replication terminates at some point despite successful target cell entry and initiation of reverse transcription. This hypothesis remains unaddressed.

## 7. Conclusion

**Figure 4.**
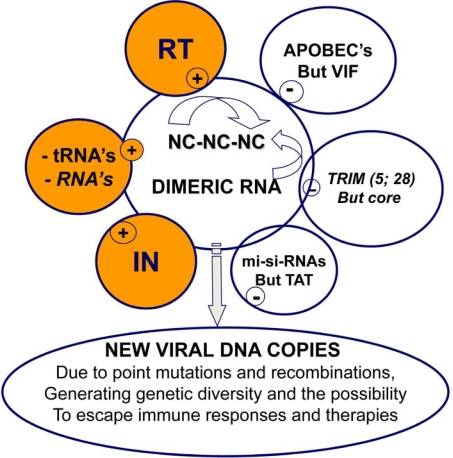
**Molecular interactions in the course of reverse transcription.** This scheme illustrates essential molecular interactions taking place prior to and during reverse transcription. (i) The genomic RNA is in a dimeric form where there are many RNA-RNA interactions, in addition to the Dimer Linkage Structure (DLS). (ii) Several hundred NC molecules, in a poorly characterized oligomeric form [72] (see top arrow pointing to NC-NC interactions), coat the genomic RNA providing protection against cellular nucleases and UV irradiation; (iii) A number of small cellular RNAs are incorporated into virions via interactions with Gag-NC and Pol-RT and Pol-IN (not illustrated here); except for the primer tRNA the function, if any, of the other cellular RNAs is poorly understood. (iv) The RT and IN enzymes interact with the genomic RNA-NC complex ensuring reverse transcription and ultimately integration of the newly made viral DNA. (v) In the absence of the viral factor VIF, APOBEC restriction factors are incorporated into virions via interactions with the viral RNA and NC, which results in the production of highly mutated viral DNA molecules. (vi) The reverse transcription machinery is housed within the incoming virion core where capsid protein molecules provide protection against host restriction factors such as TRIM proteins (see also text). (vii) Small amounts of the viral transactivator TAT have been found in the virion core. Tat may counteract the negative impact of cellular miRNA on the stability of the viral RNA prior to virion formation [124].

Studying reverse transcription in HIV-1 has shed some light on virus variability fueled by the error prone RT enzyme, which is, at least in part, counterbalanced by NC protein in order to control the process and to contribute to its completion and fidelity (Figure 4). At the same time, NC and NC-RNA interactions most probably drive the incorporation of cellular deaminase APOBEC 3G, which should in turn contribute to HIV-1 variability (Figure 4), though it in turn counterbalanced by the viral factor VIF.

To understand how HIV-1 genetic variability allows the virus to resist innate defenses, specific immunological responses and highly active antiretroviral therapies targeting the viral enzymes RT, PR and IN (HAART) [119], the circulating virus populations should be viewed as quasi-species consisting of genetically distinct but closely related viruses. Analysis of HIV-1 proviral DNA in individual T-cells present in the lymph nodes of infected persons shows a substantial fraction harbor distinct proviruses [120]. It follows that infected T-cells possess the ability to produce both homozygous and heterozygous viruses by means of recombination during reverse transcription, further fueling HIV-1 variability [18,21,121–122] and translates into the generation of a large number of virus quasi-species.

Since the nucleocapsid protein of HIV-1 plays key roles in reverse transcription, mainly through its highly conserved zinc fingers, it represents a target of choice for compounds and approaches [123] that seek to complement HAART and impair the circulation of HIV-1 strains resistant to anti-RT, -PR and – IN drugs [120].

## References

[1] Baltimore D (1970). RNA-dependent DNA polymerase in virions of RNA tumour viruses. Nature.

[2] Gilboa E, Mitra SW, Goff S, Baltimore D (1979). A detailed model of reverse transcription and tests of crucial aspects. Cell.

[3] Coffin JM, Fields BN, Knipe DM, Chanock RM, Hirsch MS, Melnick JL, Monath TP, Roizman B (1990). Retroviridae and their replication. Virology.

[4] Coffin JM (1979). Structure, replication, and recombination of retrovirus genomes: some unifying hypotheses. J Gen Virol.

[5] Mougel M, Houzet L, Darlix JL (2009). When is it time for reverse transcription to start and go?. Retrovirology.

[6] Temin HM, Mizutani S (1970). RNA-dependent DNA polymerase in virions of Rous sarcoma virus. Nature.

[7] Mizutani S, Boettiger D, Temin HM (1970). A DNA-dependent DNA polymerase and a DNA endonuclease in virions of Rous sarcoma virus. Nature.

[8] Delelis O, Carayon K, Saib A, Deprez E, Mouscadet JF (2008). Integrase and integration: biochemical activities of HIV-1 integrase. Retrovirology.

[9] Lewinski MK, Bushman FD (2005). Retroviral DNA integration--mechanism and consequences. Adv Genet.

[10] Goff SP (2007). Host factors exploited by retroviruses. Nat Rev Microbiol.

[11] Jern P, Coffin JM (2008). Effects of retroviruses on host genome function. Annu Rev Genet.

[12] Wolf D, Goff SP (2008). Host restriction factors blocking retroviral replication. Annu Rev Genet.

[13] Negre D, Duisit G, Mangeot PE, Moullier P, Darlix JL, Cosset FL (2002). Lentiviral vectors derived from simian immunodeficiency virus. Curr Top Microbiol Immunol.

[14] Beauregard A, Curcio MJ, Belfort M (2008). The take and give between retrotransposable elements and their hosts. Annu Rev Genet.

[15] Wilhelm FX, Wilhelm M, Gabriel A (2005). Reverse transcriptase and integrase of the Saccharomyces cerevisiae Ty1 element. Cytogenet Genome Res.

[16] Wilhelm M, Wilhelm FX (2001). Reverse transcription of retroviruses and LTR retrotransposons. Cell Mol Life Sci.

[17] Han JS, Boeke JD (2005). LINE-1 retrotransposons: modulators of quantity and quality of mammalian gene expression. Bioessays.

[18] Chen J, Nikolaitchik O, Singh J, Wright A, Bencsics CE, Coffin JM, Ni N, Lockett S, Pathak VK, Hu WS (2009). High efficiency of HIV-1 genomic RNA packaging and heterozygote formation revealed by single virion analysis. Proc Natl Acad Sci U S A.

[19] Chertova E, Chertov O, Coren LV, Roser JD, Trubey CM, Bess JW, Sowder RC, Barsov E, Hood BL, Fisher RJ, Nagashima K, Conrads TP, Veenstra TD, Lifson JD, Ott DE (2006). Proteomic and biochemical analysis of purified human immunodeficiency virus type 1 produced from infected monocyte-derived macrophages. J Virol.

[20] Darlix JL, Garrido JL, Morellet N, Mely Y, de Rocquigny H (2007). Properties, functions, and drug targeting of the multifunctional nucleocapsid protein of the human immunodeficiency virus. Adv Pharmacol.

[21] Darlix JL, Lapadat-Tapolsky M, de Rocquigny H, Roques BP (1995). First glimpses at structure-function relationships of the nucleocapsid protein of retroviruses. J Mol Biol.

[22] Maeda N, Fan H, Yoshikai Y (2008). Oncogenesis by retroviruses: old and new paradigms. Rev Med Virol.

[23] Iordanskiy SN, Bukrinsky MI (2009). Analysis of viral and cellular proteins in HIV-1 reverse transcription complexes by co-immunoprecipitation. Methods Mol Biol.

[24] Levin JG, Guo J, Rouzina I, Musier-Forsyth K (2005). Nucleic acid chaperone activity of HIV-1 nucleocapsid protein: critical role in reverse transcription and molecular mechanism. Prog Nucleic Acid Res Mol Biol.

[25] Rein A, Henderson LE, Levin JG (1998). Nucleic-acid-chaperone activity of retroviral nucleocapsid proteins: significance for viral replication. Trends Biochem Sci.

[26] Berkhout B (1996). Structure and function of the human immunodeficiency virus leader RNA. Prog Nucleic Acid Res Mol Biol.

[27] Kleiman L, Halwani R, Javanbakht H (2004). The selective packaging and annealing of primer tRNALys3 in HIV-1. Curr HIV Res.

[28] Molling K, Bolognesi DP, Bauer H, Busen W, Plassmann HW, Hausen P (1971). Association of viral reverse transcriptase with an enzyme degrading the RNA moiety of RNA-DNA hybrids. Nat New Biol.

[29] Klarmann GJ, Hawkins ME, Le Grice SF (2002). Uncovering the complexities of retroviral ribonuclease H reveals its potential as a therapeutic target. AIDS Rev.

[30] Allain B, Lapadat TM, Berlioz C, Darlix JL (1994). Transactivation of the minus-strand DNA transfer by nucleocapsid protein during reverse transcription of the retroviral genome. Embo J.

[31] Munch J, Rucker E, Standker L, Adermann K, Goffinet C, Schindler M, Wildum S, Chinnadurai R, Rajan D, Specht A, Gimenez-Gallego G, Sanchez PC, Fowler DM, Koulov A, Kelly JW, Mothes W, Grivel JC, Margolis L, Keppler OT, Forssmann WG, Kirchhoff F (2007). Semen-derived amyloid fibrils drastically enhance HIV infection. Cell.

[32] Tsuchihashi Z, Brown PO (1994). DNA strand exchange and selective DNA annealing promoted by the human immunodeficiency virus type 1 nucleocapsid protein. J Virol.

[33] Godet J, de Rocquigny H, Raja C, Glasser N, Ficheux D, Darlix JL, Mely Y (2006). During the early phase of HIV-1 DNA synthesis, nucleocapsid protein directs hybridization of the TAR complementary sequences via the ends of their double-stranded stem. J Mol Biol.

[34] Lener D, Tanchou V, Roques BP, Le Grice SF, Darlix JL (1998). Involvement of HIV-I nucleocapsid protein in the recruitment of reverse transcriptase into nucleoprotein complexes formed *in vitro*. J Biol Chem.

[35] Jacob DT, DeStefano JJ (2008). A new role for HIV nucleocapsid protein in modulating the specificity of plus strand priming. Virology.

[36] Post K, Kankia B, Gopalakrishnan S, Yang V, Cramer E, Saladores P, Gorelick RJ, Guo J, Musier-Forsyth K, Levin JG (2009). Fidelity of plus-strand priming requires the nucleic acid chaperone activity of HIV-1 nucleocapsid protein. Nucleic Acids Res.

[37] Abbondanzieri EA, Bokinsky G, Rausch JW, Zhang JX, Le Grice SF, Zhuang X (2008). Dynamic binding orientations direct activity of HIV reverse transcriptase. Nature.

[38] Auxilien S, Keith G, Le Grice SF, Darlix JL (1999). Role of post-transcriptional modifications of primer tRNALys,3 in the fidelity and efficacy of plus strand DNA transfer during HIV-1 reverse transcription. J Biol Chem.

[39] Ramalanjaona N, de Rocquigny H, Millet A, Ficheux D, Darlix JL, Mely Y (2007). Investigating the mechanism of the nucleocapsid protein chaperoning of the second strand transfer during HIV-1 DNA synthesis. J Mol Biol.

[40] Buckman JS, Bosche WJ, Gorelick RJ (2003). Human immunodeficiency virus type 1 nucleocapsid zn(2+) fingers are required for efficient reverse transcription, initial integration processes, and protection of newly synthesized viral DNA. J Virol.

[41] Carteau S, Gorelick RJ, Bushman FD (1999). Coupled integration of human immunodeficiency virus type 1 cDNA ends by purified integrase *in vitro*: stimulation by the viral nucleocapsid protein. J Virol.

[42] Ciuffi A, Bushman FD (2006). Retroviral DNA integration: HIV and the role of LEDGF/p75. Trends Genet.

[43] Ciuffi A, Llano M, Poeschla E, Hoffmann C, Leipzig J, Shinn P, Ecker JR, Bushman F (2005). A role for LEDGF/p75 in targeting HIV DNA integration. Nat Med.

[44] Di Santo R, Costi R, Roux A, Miele G, Crucitti GC, Iacovo A, Rosi F, Lavecchia A, Marinelli L, Di Giovanni C, Novellino E, Palmisano L, Andreotti M, Amici R, Galluzzo CM, Nencioni L, Palamara AT, Pommier Y, Marchand C (2008). Novel quinolinonyl diketo acid derivatives as HIV-1 integrase inhibitors: design, synthesis, and biological activities. J Med Chem.

[45] Campbell S, Vogt VM (1995). Self-assembly *in vitro* of purified CA-NC proteins from Rous sarcoma virus and human immunodeficiency virus type 1. J Virol.

[46] Muriaux D, Mirro J, Harvin D, Rein A (2001). RNA is a structural element in retrovirus particles. Proc Natl Acad Sci U S A.

[47] Zuber G, McDermott J, Karanjia S, Zhao W, Schmid MF, Barklis E (2000). Assembly of retrovirus capsid-nucleocapsid proteins in the presence of membranes or RNA. J Virol.

[48] Liang C, Hu J, Russell RS, Kameoka M, Wainberg MA (2004). Spliced human immunodeficiency virus type 1 RNA is reverse transcribed into cDNA within infected cells. AIDS Res Hum Retroviruses.

[49] Houzet L, Paillart JC, Smagulova F, Maurel S, Morichaud Z, Marquet R, Mougel M (2007). HIV controls the selective packaging of genomic, spliced viral and cellular RNAs into virions through different mechanisms. Nucleic Acids Res.

[50] Houzet L, Morichaud Z, Mougel M (2007). Fully-spliced HIV-1 RNAs are reverse transcribed with similar efficiencies as the genomic RNA in virions and cells, but more efficiently in AZT-treated cells. Retrovirology.

[51] Maurel S, Houzet L, Garcia EL, Telesnitsky A, Mougel M (2007). Characterization of a natural heterodimer between MLV genomic RNA and the SD' retroelement generated by alternative splicing. RNA.

[52] Berkowitz R, Fisher J, Goff SP (1996). RNA packaging. Curr Top Microbiol Immunol.

[53] Katz RA, Terry RW, Skalka AM (1986). A conserved cis-acting sequence in the 5′ leader of avian sarcoma virus RNA is required for packaging. J Virol.

[54] Russell RS, Roldan A, Detorio M, Hu J, Wainberg MA, Liang C (2003). Effects of a single amino acid substitution within the p2 region of HIV-1 on packaging of spliced viral RNA. J Virol.

[55] Zhang Y, Barklis E (1995). Nucleocapsid protein effects on the specificity of retrovirus RNA encapsidation. J Virol.

[56] Housset V, Darlix JL (1996). Mutations at the capsid-nucleocapsid cleavage site of gag polyprotein of Moloney murine leukemia virus abolish virus infectivity. C R Acad Sci III.

[57] Sinck L, Richer D, Howard J, Alexander M, Purcell DF, Marquet R, Paillart JC (2007). *In vitro* dimerization of human immunodeficiency virus type 1 (HIV-1) spliced RNAs. RNA.

[58] Houzet L, Battini J, Bernard E, Thibert V, Mougel M (2003). A new retroelement constituted by a natural alternatively spliced RNA of murine replication-competent retroviruses. EMBO J.

[59] Adkins B, Hunter T (1981). Identification of a packaged cellular mRNA in virions of rous sarcoma virus. J Virol.

[60] Aronoff R, Linial M (1991). Specificity of retroviral RNA packaging. J Virol.

[61] Ikawa Y, Ross J, Leder P (1974). An association between globin messenger RNA and 60S RNA derived from Friend leukemia virus. Proc Natl Acad Sci U S A.

[62] Gallis B, Linial M, Eisenman R (1979). An avian oncovirus mutant deficient in genomic RNA: characterization of the packaged RNA as cellular messenger RNA. Virology.

[63] Linial M, Medeiros E, Hayward WS (1978). An avian oncovirus mutant (SE 21Q1b) deficient in genomic RNA: biological and biochemical characterization. Cell.

[64] Rulli SJ, Hibbert CS, Mirro J, Pederson T, Biswal S, Rein A (2007). Selective and nonselective packaging of cellular RNAs in retrovirus particles. J Virol.

[65] Onafuwa-Nuga AA, King SR, Telesnitsky A (2005). Nonrandom packaging of host RNAs in moloney murine leukemia virus. J Virol.

[66] Tian C, Wang T, Zhang W, Yu XF (2007). Virion packaging determinants and reverse transcription of SRP RNA in HIV-1 particles. Nucleic Acids Res.

[67] Giles KE, Caputi M, Beemon KL (2004). Packaging and reverse transcription of snRNAs by retroviruses may generate pseudogenes. RNA.

[68] Garcia EL, Onafuwa-Nuga A, Sim S, King SR, Wolin SL, Telesnitsky A (2009). Packaging of host mY RNAs by murine leukemia virus may occur early in Y RNA biogenesis. J Virol.

[69] Onafuwa-Nuga A, Telesnitsky A (2009). The remarkable frequency of human immunodeficiency virus type 1 genetic recombination. Microbiol Mol Biol Rev.

[70] Kleiman L (2002). tRNA(Lys3): the primer tRNA for reverse transcription in HIV-1. IUBMB Life.

[71] Levin JG, Seidman JG (1979). Selective packaging of host tRNA's by murine leukemia virus particles does not require genomic RNA. J Virol.

[72] Darlix JL, Cristofari G, Rau M, Pechoux C, Berthoux L, Roques B (2000). Nucleocapsid protein of human immunodeficiency virus as a model protein with chaperoning functions and as a target for antiviral drugs. Adv Pharmacol.

[73] Strebel K, Khan MA (2008). APOBEC3G encapsidation into HIV-1 virions: which RNA is it. Retrovirology.

[74] Wang T, Tian C, Zhang W, Sarkis PT, Yu XF (2008). Interaction with 7SL RNA but not with HIV-1 genomic RNA or P bodies is required for APOBEC3F virion packaging. J Mol Biol.

[75] D'Souza V, Summers MF (2005). How retroviruses select their genomes. Nat Rev Microbiol.

[76] Cimarelli A, Darlix JL (2002). Assembling the human immunodeficiency virus type 1. Cell Mol Life Sci.

[77] Freed EO (2002). Viral late domains. J Virol.

[78] Freed EO (1998). HIV-1 gag proteins: diverse functions in the virus life cycle. Virology.

[79] Ganser-Pornillos BK, Yeager M, Sundquist WI (2008). The structural biology of HIV assembly. Curr Opin Struct Biol.

[80] Briggs JA, Riches JD, Glass B, Bartonova V, Zanetti G, Krausslich HG (2009). Structure and assembly of immature HIV. Proc Natl Acad Sci U S A.

[81] Briggs JA, Simon MN, Gross I, Krausslich HG, Fuller SD, Vogt VM, Johnson MC (2004). The stoichiometry of Gag protein in HIV-1. Nat Struct Mol Biol.

[82] Raposo G, Moore M, Innes D, Leijendekker R, Leigh-Brown A, Benaroch P, Geuze H (2002). Human macrophages accumulate HIV-1 particles in MHC II compartments. Traffic.

[83] Basyuk E, Galli T, Mougel M, Blanchard JM, Sitbon M, Bertrand E (2003). Retroviral genomic RNAs are transported to the plasma membrane by endosomal vesicles. Dev Cell.

[84] Grigorov B, Arcanger F, Roingeard P, Darlix JL, Muriaux D (2006). Assembly of infectious HIV-1 in human epithelial and T-lymphoblastic cell lines. J Mol Biol.

[85] Houzet L, Gay B, Morichaud Z, Briant L, Mougel M (2006). Intracellular assembly and budding of the Murine Leukemia Virus in infected cells. Retrovirology.

[86] Darlix JL, Bromley PA, Spahr PF (1977). New procedure for the direct analysis of *in vitro* reverse transcription of Rous sarcoma virus RNA. J Virol.

[87] Lori F, di Marzo Veronese F, de Vico AL, Lusso P, Reitz MS, Gallo RC (1992). Viral DNA carried by human immunodeficiency virus type 1 virions. J Virol.

[88] Trono D (1992). Partial reverse transcripts in virions from human immunodeficiency and murine leukemia viruses. J Virol.

[89] Zhang Y, Qian H, Love Z, Barklis E (1998). Analysis of the assembly function of the human immunodeficiency virus type 1 gag protein nucleocapsid domain. J Virol.

[90] Zhang H, Dornadula G, Pomerantz RJ (1996). Endogenous reverse transcription of human immunodeficiency virus type 1 in physiological microenviroments: an important stage for viral infection of nondividing cells. J Virol.

[91] Roan NR, Greene WC (2007). A seminal finding for understanding HIV transmission. Cell.

[92] Rothenberg E, Smotkin D, Baltimore D, Weinberg RA (1977). *In vitro* synthesis of infectious DNA of murine leukaemia virus. Nature.

[93] Borroto-Esoda K, Boone LR (1991). Equine infectious anemia virus and human immunodeficiency virus DNA synthesis *in vitro*: characterization of the endogenous reverse transcriptase reaction. J Virol.

[94] Roan NR, Sowinski S, Munch J, Kirchhoff F, Greene WC (2010). Aminoquinoline surfen inhibits the action of SEVI (semen-derived enhancer of viral infection). J Biol Chem.

[95] Thomas JA, Gorelick RJ (2008). Nucleocapsid protein function in early infection processes. Virus Res.

[96] Houzet L, Morichaud Z, Didierlaurent L, Muriaux D, Darlix JL, Mougel M (2008). Nucleocapsid mutations turn HIV-1 into a DNA-containing virus. Nucleic Acids Res.

[97] Didierlaurent L, Houzet L, Morichaud Z, Darlix JL, Mougel M (2008). The conserved N-terminal basic residues and zinc-finger motifs of HIV-1 nucleocapsid restrict the viral cDNA synthesis during virus formation and maturation. Nucleic Acids Res.

[98] Thomas JA, Bosche WJ, Shatzer TL, Johnson DG, Gorelick RJ (2008). Mutations in human immunodeficiency virus type 1 nucleocapsid protein zinc fingers cause premature reverse transcription. J Virol.

[99] Grigorov B, Decimo D, Smagulova F, Pechoux C, Mougel M, Muriaux D, Darlix JL (2007). Intracellular HIV-1 Gag localization is impaired by mutations in the nucleocapsid zinc fingers. Retrovirology.

[100] Brun S, Solignat M, Gay B, Bernard E, Chaloin L, Fenard D, Devaux C, Chazal N, Briant L (2008). VSV-G pseudotyping rescues HIV-1 CA mutations that impair core assembly or stability. Retrovirology.

[101] Dismuke DJ, Aiken C (2006). Evidence for a functional link between uncoating of the human immunodeficiency virus type 1 core and nuclear import of the viral preintegration complex. J Virol.

[102] Forshey BM, von Schwedler U, Sundquist WI, Aiken C (2002). Formation of a human immunodeficiency virus type 1 core of optimal stability is crucial for viral replication. J Virol.

[103] Qi M, Aiken C (2008). Nef enhances HIV-1 infectivity via association with the virus assembly complex. Virology.

[104] Arhel NJ, Souquere-Besse S, Munier S, Souque P, Guadagnini S, Rutherford S, Prevost MC, Allen TD, Charneau P (2007). HIV-1 DNA Flap formation promotes uncoating of the pre-integration complex at the nuclear pore. EMBO J.

[105] Saphire AC, Guan T, Schirmer EC, Nemerow GR, Gerace L (2000). Nuclear import of adenovirus DNA *in vitro* involves the nuclear protein import pathway and hsc70. J Biol Chem.

[106] Ojala PM, Sodeik B, Ebersold MW, Kutay U, Helenius A (2000). Herpes simplex virus type 1 entry into host cells: reconstitution of capsid binding and uncoating at the nuclear pore complex *in vitro*. Mol Cell Biol.

[107] Rabe B, Delaleau M, Bischof A, Foss M, Sominskaya I, Pumpens P, Cazenave C, Castroviejo M, Kann M (2009). Nuclear entry of hepatitis B virus capsids involves disintegration to protein dimers followed by nuclear reassociation to capsids. PLoS Pathog.

[108] Stremlau M, Owens CM, Perron MJ, Kiessling M, Autissier P, Sodroski J (2004). The cytoplasmic body component TRIM5alpha restricts HIV-1 infection in Old World monkeys. Nature.

[109] Auewarakul P, Wacharapornin P, Srichatrapimuk S, Chutipongtanate S, Puthavathana P (2005). Uncoating of HIV-1 requires cellular activation. Virology.

[110] Miller MD, Warmerdam MT, Ferrell SS, Benitez R, Greene WC (1997). Intravirion generation of the C-terminal core domain of HIV-1 Nef by the HIV-1 protease is insufficient to enhance viral infectivity. Virology.

[111] Riviere L, Darlix JL, Cimarelli A Analysis of the viral elements required in the nuclear import of HIV-1 DNA. J Virol.

[112] Manganini M, Serafini M, Bambacioni F, Casati C, Erba E, Follenzi A, Naldini L, Bernasconi S, Gaipa G, Rambaldi A, Biondi A, Golay J, Introna M (2002). A human immunodeficiency virus type 1 pol gene-derived sequence (cPPT/CTS) increases the efficiency of transduction of human nondividing monocytes and T lymphocytes by lentiviral vectors. Hum Gene Ther.

[113] Dvorin JD, Bell P, Maul GG, Yamashita M, Emerman M, Malim MH (2002). Reassessment of the roles of integrase and the central DNA flap in human immunodeficiency virus type 1 nuclear import. J Virol.

[114] Mbisa JL, Delviks-Frankenberry KA, Thomas JA, Gorelick RJ, Pathak VK (2009). Real-time PCR analysis of HIV-1 replication post-entry events. Methods Mol Biol.

[115] Arfi V, Lienard J, Nguyen XN, Berger G, Rigal D, Darlix JL, Cimarelli A (2009). Characterization of the behavior of functional viral genomes during the early steps of human immunodeficiency virus type 1 infection. J Virol.

[116] Arfi V, Riviere L, Jarrosson-Wuilleme L, Goujon C, Rigal D, Darlix JL, Cimarelli A (2008). Characterization of the early steps of infection of primary blood monocytes by human immunodeficiency virus type 1. J Virol.

[117] Skasko M, Weiss KK, Reynolds HM, Jamburuthugoda V, Lee K, Kim B (2005). Mechanistic differences in RNA-dependent DNA polymerization and fidelity between murine leukemia virus and HIV-1 reverse transcriptases. J Biol Chem.

[118] Thomas BE, Ramachandran R, Anitha S, Swaminathan S (2007). Feasibility of routine HIV testing among TB patients through a voluntary counselling and testing centre. Int J Tuberc Lung Dis.

[119] HIV drug resistant mutations by drug class. Stanford HIV Drug resistance database. http://hivdb.stanford.edu.

[120] Bocharov G, Ford NJ, Edwards J, Breinig T, Wain-Hobson S, Meyerhans A (2005). A genetic-algorithm approach to simulating human immunodeficiency virus evolution reveals the strong impact of multiply infected cells and recombination. J Gen Virol.

[121] Hu WS, Temin HM (1990). Genetic consequences of packaging two RNA genomes in one retroviral particle: pseudodiploidy and high rate of genetic recombination. Proc. Natl. Acad. Sci. USA.

[122] Baird HA, Gao Y, Galetto R, Lalonde M, Anthony RM, Giacomoni V, Abreha M, Destefano JJ, Negroni M, Arts EJ (2006). Influence of sequence identity and unique breakpoints on the frequency of intersubtype HIV-1 recombination. Retrovirology.

[123] Goldschmidt V, Lisa M, Jenkins L, de Rocquigny H, Darlix JL, Mély Y (2010). The nucleocapsid protein of HIV-1 as a promising therapeutic target for antiviral drugs. HIV Therapy.

[124] Bennasser Y, Yeung ML, Benkirane M, Jeang KT (2006). RNA interference and HIV-1: hits and misses. Curr Opin HIV AIDS.

